# The prognostic relevance of a gene expression signature in MRI-defined highly vascularized glioblastoma

**DOI:** 10.1016/j.heliyon.2024.e31175

**Published:** 2024-05-17

**Authors:** Víctor Montosa-i-Micó, María del Mar Álvarez-Torres, Rebeca Burgos-Panadero, F. Javier Gil-Terrón, Maria Gómez Mahiques, Carles Lopez-Mateu, Juan M. García-Gómez, Elies Fuster-Garcia

**Affiliations:** a Instituto Universitario de Tecnologías de la Información y Comunicaciones (ITACA), BDSLab, Universitat Politècnica de València, Spain; b Laboratory of Cellular and Molecular Biology, Clinical and Translational Research in Cancer Group, La Fe Health Research Institute, Valencia, Spain

**Keywords:** High-grade glioma, Glioblastoma, Relative cerebral blood volume, Tumor vascularity, RNA-seq, ONCOhabitats, Biomarker, MRI

## Abstract

**Background:**

The vascular heterogeneity of glioblastomas (GB) remains an important area of research, since tumor progression and patient prognosis are closely tied to this feature. With this study, we aim to identify gene expression profiles associated with MRI-defined tumor vascularity and to investigate its relationship with patient prognosis.

**Methods:**

The study employed MRI parameters calculated with DSC Perfusion Quantification of ONCOhabitats glioma analysis software and RNA-seq data from the TCGA-GBM project dataset. In our study, we had a total of 147 RNA-seq samples, which 15 of them also had MRI parameter information. We analyzed the gene expression profiles associated with MRI-defined tumor vascularity using differential gene expression analysis and performed Log-rank tests to assess the correlation between the identified genes and patient prognosis.

**Results:**

The findings of our research reveal a set of 21 overexpressed genes associated with the high vascularity pattern. Notably, several of these overexpressed genes have been previously implicated in worse prognosis based on existing literature. Our log-rank test further validates that the collective upregulation of these genes is indeed correlated with an unfavorable prognosis. This set of genes includes a variety of molecules, such as cytokines, receptors, ligands, and other molecules with diverse functions.

**Conclusions:**

Our findings suggest that the set of 21 overexpressed genes in the High Vascularity group could potentially serve as prognostic markers for GB patients. These results highlight the importance of further investigating the relationship between the molecules such as cytokines or receptors underlying the vascularity in GB and its observation through MRI and developing targeted therapies for this aggressive disease.

## Highlights

• Identified 21 genes linked to GB MRI vascularity and poor prognosis.
• Prediction of GB transcriptomic profile from MRI vascularity.
• MRI-based vascularity profiles can predict GB patient survival.
• Gene set offers new GB biomarkers for clinical assessment.

## 1 Introduction & background

Glioblastoma (GB) is the most common malignant primary brain tumor, representing approximately 57 % of all gliomas, and 48 % of all primary malignant central nervous system (CNS) tumors [[1], [2], [3], [4]]. The overall prevalence of GB in the United States is 9.23 per 100,000 population. GB's prognosis is very poor, with a median survival of 1.5-2-year treatment schedule. Due to their infiltrative nature, GBs are resistant to conventional therapies and hinders full isolation of tumor in aggressive surgical removals [5]. Standard treatment of GB includes surgery, radiotherapy, and temozolomide chemotherapy treatment [6].

Vascularization and angiogenesis play a significant role in driving the progression of GB [7]. Tumor tissues that receive better irrigation can obtain a larger supply of metabolites required for anaerobic glycolysis, which is a characteristic of tumor metabolism with high rates of glucose consumption. This process accelerates cell proliferation and tumor growth [8].

Vascularization and angiogenesis, which are the physiological processes that determine to a greater extent the vascularity of a tumor can be characterized from the analysis of the transcriptome of those genes involved in processes related to vascularity. From RNA-seq data, we can assess the heterogeneity present in the transcriptome among GB patients, as well as changes in splicing patterns, post-translational modifications, or RNA sequence alterations caused by mutations or polymorphisms present in the DNA exome [9,10]. The analysis of these bioinformatics data can be very useful, but is impractical in the clinic for each patient, as it involves high economic costs, time and effort.

MRI-derived perfusion parameters are directly associated with the tumor vascularity in glioblastoma, providing information on blood flow, vascular permeability, and nutrient and oxygen exchange capacity [11]. This correlation may prove valuable for the management of this highly malignant brain neoplasm [12].

Linking transcriptomic vascular profiles with biomarkers that can be calculated non-invasively and pre-surgically, such as MRI perfusion parameters, could revolutionize the treatment of GB by providing not only the macroscopic information extractable from the MRI itself, but also the molecular biology information of the vascular profile associated with certain values of the MRI parameters. Specifically, establishing an association between the expression of a group of genes and macroscopic vascularity would improve the pre-surgical evaluation of GB patients [13]. To this end, researchers have investigated the use of differential perfusion parameters, such as relative cerebral blood volume (rCBV), which has been extensively analyzed in the literature as an accurate prognostic marker [[14], [15], [16]]. The rCBV can be noninvasively obtained at every stage of the tumor.

This study aims to examine that the macroscopic manifestations of vascularity described by MRI parameters and certain transcriptomes are associated. The main objectives are to obtain a minimum significant set of vascularity-related genes that represent the different transcriptome patterns and investigate its correlation with patient prognosis.

## 2 Materials & methods

### 2.1 Patients’ cohort

The database used for this study is the TCGA-GBM (https://portal.gdc.cancer.gov/projects/TCGA-GBM) [17], includes data from 617 patients. From those patients, we included only those with RNA-seq data.

The criteria for including the TCGA RNA-seq samples was to select those with the label “Primary Tumor”. For cases with more than one sample, one was chosen randomly. TCGA IDs can be found in Additional File 1. Patients with damaged or incomplete RNA-seq or MRI data have been duly ruled out. MRI series are available at The Cancer Imaging Archive (TCIA): https://wiki.cancerimagingarchive.net/pages/viewpage.action?pageId=1966258. The definition of group criteria was influenced by the lack of MRI perfusion sequences and RNA-seq data for all patients. In the Results section, a table containing information on the variables of sex, race, and molecular tumor subtype was included to provide details about the total sample used in the study.

### 2.2 RCBV calculation from MRIs

RCBV is calculated using DSC-MRI, which involves capturing T2*-weighted images at high temporal resolution during a bolus injection of a gadolinium-based contrast agent (GBCA). This technique estimates rCBV by analyzing the change in the relaxation rate (ΔR2 * (t)) of the brain tissue caused by the contrast agent, which is confined to the blood vessels [18]. For the quantification of the rCBV measure of each patient, we used the ONCOhabitats platform (publicly accessible at ONCOhabitats site: https://www.oncohabitats.upv.es), which provides an automated unsupervised method to describe the heterogeneity of the enhancing tumor and edema tissues of high-grade gliomas. The methodology consists of 4 phases: 1) Preprocessing, where most artifacts and noise are corrected and additionally, automated registration, brain extraction and intensity normalization are conducted to generate a consistent multi-parametric high-quality MRI of the brain; 2) Segmentation: tumor tissues are delimited from a 3D convolutional neural network (CNN) classifier based on a U-Net architecture. 3) Quantification of the hemodynamic indices derived from the dynamic susceptibility contrast perfusion sequence is then performed. In addition to cerebral blood flow (CBF), mean transit time (MTT), and cerebral blood volume (CBV) is calculated. 4) Hemodynamic tissue signature: ONCOhabitats classifies GBs into four distinct sub-compartments or “habitats” based on their morphological and hemodynamic characteristics. These habitats are determined by ONCOhabitats using specific criteria. The four identified habitats within the tumor and the surrounding edema are as follows: the high-angiogenic tumor (HAT) (the more perfused area of the enhancing tumor), the low-angiogenic tumor (LAT) (the area of the enhancing tumor with a lower angiogenic profile), the potentially infiltrated peripheral edema (IPE) (the surrounding non-enhancing region adjacent to the tumor with elevated perfusion indexes), and the vasogenic peripheral edema (VPE) (the remaining edema with a lower perfusion profile) [19,20]. ONCOhabitats calculates a rCBV value for each tumor region.

For this study, only the rCBV data from the HAT region (rCBV_HAT_) were collected since it presents a higher association with patient OS [21,22]. The rCBV_HAT_ median parameter calculated from the values of each pixel in the HAT habitat has been used in this study in order to define the high and moderate vascular groups according to MRI perfusion parameters.

### 2.3 Definition of vascular groups

The study cohort was subdivided in the following three groups according to their vascularity as defined by DSC-MRI perfusion.

• High Vascularity group (HV): Includes the data of patients who exceed the threshold of rCBV _HAT_ = 7.
• Moderate Vascularity group (MV): Includes the data of patients who did not reach the threshold of rCBV_HAT_ = 7.
• Unknown Vascularity group (-V): Includes data from patients for whom rCBV parameters are not known due to a lack of MRI data.

### 2.4 Gene selection criteria

Gene Ontology (GO) [23] was used to filter and select for this study only those genes with an involvement in the development and formation of new blood vessels. The filter contained the following terms: “vascular”, “vasculature”, “blood vessel”, “microvessel”, “angiogenesis”, “hypoxia”, “necrosis”, “capillary”, “glomerulus”, “glomerular”, “vasculogenesis”, “venous”, “artery”, “circulatory”. “coagulation”. Note that terms like “neovascularization” are included in “vascular".

### 2.5 Normalization of the expression matrix and low expression exclusion

We did a normalization considering the library size: this normalization consists of dividing the expression of a gene in a sample by the sum of the expression of all the genes in the same sample and multiplying it by one million to obtain values in Counts per Million (CPM). Genes that did not exceed CPM_median_ < 0.2 were excluded because they were more subject to error due to low expression [24].

### 2.6 Differential gene expression analysis

A pipeline based on the voom and limma functions has been used to carry out the differential gene expression analysis between groups according to their tumor vascularization. Those genes with very high counts have very little variability and vice versa. The mean variance trend is used to build the regression models for each gene. Voom learns this trend and obtains weights for each gene and sample that are passed into limma along with the log2 (using the CPM normalized values). With limma, we built a regression model for each gene, in which the expression was used as the dependent variable and the cohort as the independent variable. In the voom function associated with the results of the limma function, we generated a model with age and cohort as independent variables [25,26].

For the hypothesis tests, we utilized a modified version of the limma model that incorporated Empirical Bayes smoothing of standard errors [27]. This technique was used to adjust standard errors that deviated greatly from those of other genes toward the average standard error.

It has been assigned a significance level of Q value < 0.01 (considering False Discovery Rate [28]) to determine that the gene expression between groups was significantly different or not. Hypothesis tests were developed based on the log fold changes (logFC) obtained from the differential gene expression analysis between the MV or HV group and the -V group. A positive LogFC means overexpression in the MV or HV group with respect to the -V group and vice versa.

Volcano plots were made to visualize the statistical significance and the magnitude of the change in gene expression between the two groups with vascularity defined with the -V group.

### 2.7 Correlation between differentially expressed genes and prognosis

A score system evaluated gene expression and vascularity's correlation with survival rates. Kaplan-Meier curves and Log-Rank test were used to analyze the relationship between prognosis and gene expression.

### 2.8 The score system

The Score system compares the expression level of each differentially expressed gene with the average expression across all samples. Samples with higher expression than the average received +1 point if the gene had higher expression than the -V group, and samples with lower expression received +1 point if the gene had lower expression than the -V group. No points were given in any other cases. This process was repeated for every sample to calculate a total score. The same process was repeated for each RNA-seq sample in the gene expression study, thus obtaining a simple quantification of the expression of vascularity genes in all patients and thus defining the vascularity of the patients, including those in group -V (without vascularity defined by MRI parameters).

**Figure:**
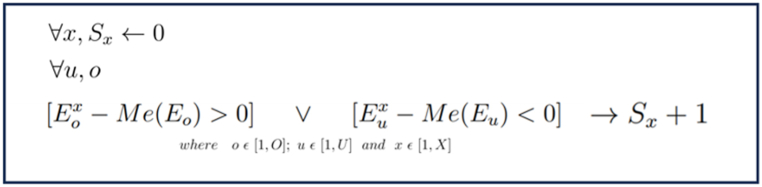

Algorithm 1 Algorithm explaining our Score system: S = Score, X = Number of samples E = Expression, Me = Median, O = number of overexpressed genes in the DGE analysis, U = Number of Underexpressed genes in the DGE analysis.

### 2.9 Kaplan-Meier group definition

Two Kaplan-Meier curve analyses were proposed, one for each differential expression analysis (HV vs -V and MV vs -V). A high score meant high similarity to the gene expression pattern faced in each differential expression analysis. Therefore, for each Kaplan-Meier curve analysis, the Expected High Vascularity (EHV) and the Expected Moderate Vascularity (EMV) groups were defined in opposite ways, according to Table 1 below.

**Table 1:**
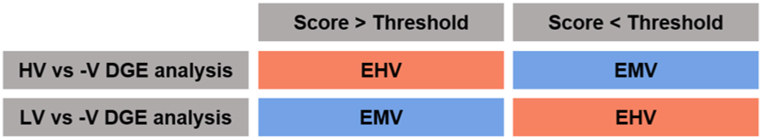
Defining the groups according to each DGE analysis. EHV = Expected High Vascularity group, EMV = Expected Moderate Vascularity group.

The threshold for differentiating patients according to the value in the score system was the one that yields maximal difference with regard to survival between the two groups at the lowest log-rank *P*-value.

## 3 Results

### 3.1 Patients’ cohort

Table 2 presents the clinical characteristics of the patient cohort, with no significant differences in survival among tumor subtypes, genders, or races as indicated by the log-rank test p-values.

**Table 2:** Patient cohort clinical details. *P*-values from log-rank tests comparing survival distributions across groups.

Variable	Data	p-value
Verhaak Tumor Subtype	109	0.835
Classical	29	
Mesenchymal	37	
Neural	19	
Proneural	24	
Gender	146	0.74
Male	94	
Female	52	
Race	145	0.4
Asian	3	
Black or African American	10	
White	132	
Age	142	0.001*
Q1 [21.44 53.31]	36	
Q2 [53.31–62.6]	35	
Q3 [62.6–70.63]	35	
Q4 [70.63–89.35]	36	

### 3.2 Definition of vascular groups

**Fig. 1:**
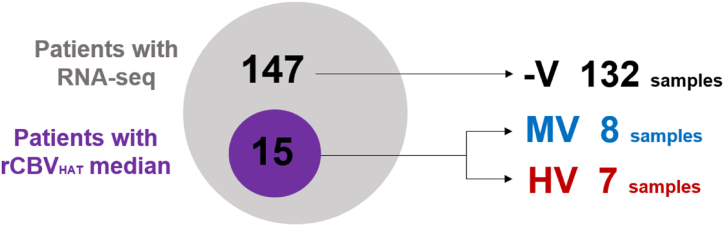
Venn diagram explaining how our sample is organized. rCBV_HAT_ = Relative Cerebral Blood Flow at High Angiogenic Tumor habitat. -V = Undefined Vascularity Group, MV = Moderate Vascularity group, HV = High Vascularity Group.

In this study, RNA-seq data from 147 patients with GB were analyzed. However, only DSC-MRI studies were available for 15 patients. The 147 patients were divided into three groups based on the MRI available data. The first group called -V, consisted of 132 patients with RNA-seq data but no information on MRI vascularity. The remaining two groups were defined based on the rCBV_HAT_ median parameter and included 8 patients in the MV group and 7 patients in the HV group. The average rCBV_HAT_ value was 4.17 for the MV group and 8.31 for the HV group. The minimum and maximum rCBV_HAT_ values for the MV group were 2.23 and 5.92, respectively, while for the HV group, they were 7.45 and 9.32 (see Fig. 1).

### 3.3 Genes selection criteria results

**Fig. 2:**
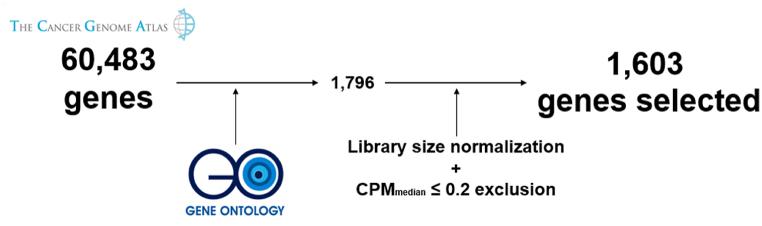
Gene selection criteria for transcriptomic profiling analysis. Gene Ontology filter is used to select the genes with an involvement in the development and formation of new blood vessels. Library size normalization is applied to adjust the number of sequences in each library to a common or standard size. CPM_median_ = Counts Per Million median of the gene. CPM_median_ ≤ 0.2 exclusion has been applied to exclude genes with insufficient expression and low informativity.

The GO filter in which all those genes with terms related to vascularity in their description were included resulted in a reduction of 60,483 genes to 1796. CPMmedian ≤0.2 exclusion reduces the selection to 1603 genes, 2.65 % of the initial genes (see Fig. 2).

### 3.4 Differential gene expression analysis – volcano plot

**Fig. 3:**
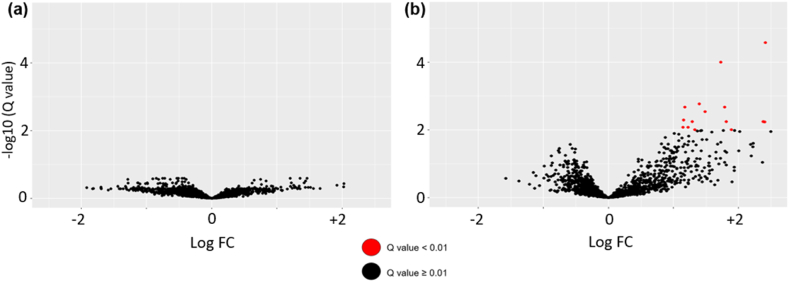
Volcano Plots of (a) MV vs -V and (b) HV vs -V gene expression. If log FC is positive, the gene has higher expression than in the -V group and vice versa.

The volcano plots in Fig. 3 illustrate the differential gene expression analysis. MV and HV groups have been compared with the -V group. Regarding the -V group, although the sample has an indefinite vascularity and the range of vascularity may be wide, it is expected that the group represents an average vascularity due to its large sample size (132 patients). In the MV group (Fig. 3a) we did not find any differentially expressed genes. By contrast, we found 21 genes significantly overexpressed for the HV group (Fig. 3b), with a restrictive Q-value significance level of 0.01.

In Table 3, we can observe in more detail the logFC of the expression of each overexpressed gene and its adjusted *P*-value. In addition, we can see that most of the genes found in our results have been shown in other studies to have a negative correlation with prognosis. Proof of this are the *P*-values of the log-rank test of The Human Protein Atlas (THPA) [29], (note that the result shown is for the cut-off that separates the sample of patients from the TCGA to obtain the most significant *P*-value), where 19 of the 31 have a Q-value <0.01, and other evidence that we have found in the literature, concretely for 23 of the 31 genes in our results.

**Table 3:** Overexpressed genes in HV group with adjusted p-value <0.01 showed in Fig. 4b. LogFC = Log fold changes of HV/-V gene expression quotient. adj P Val = Adjusted *P*-value considering False Discovery Rate [18]. UniProtKB = UniProtKB molecular function summary (CCR: C–C Chemokine Receptor; MHC: Major Histocompatibility Comple; PRR: Pattern Recognition Receptor; LPS: Lipopolysaccharide). THPA = The Human Protein Atlas Kaplan-Meier *P*-value results at best cutoff, ‘*’ means *P*-value <0.05, ‘***’ means P value < 0.01. Prognosis findings = scientific evidence relating to the correlation of overexpressed genes with a worse prognosis, ‘***’ means that we consider it is especially interesting.

	DGE ANALYSIS DATA		UniProtKB	LITERATURE PROGNOSIS DATA	
GENE	logFC	q-Value		THPA	Prognosis findings
CCL4	2.673	0	- CCR1/5 binding - Identical protein binding - Chemokine - Cytokine	0.22	Hypoxic macrophages show greater pro-invasion effect through the elevated secretion of CCL4 [30]
CCL3	2.676	0.00002	- CCR1/5 binding - Identical protein binding - Chemokine - Cytokine - Kinase - Phospholipase	0.16	no relevant info
IL10	2.413	0.00003	- Cytokine - Growth factor - Dimerization	0.2	IL10 plays crucial immunosuppressive roles in GB, thereby promoting tumor progression and immune evasion [31].
C5AR2	1.73	0.0001	- G-protein coupled receptor - Complement component C5a receptor	0.33	no relevant info
IL6	2.666	0.00041	- Cytokine - Growth factor - Dimerization	0.045*	Disruption of IL-6 signaling in HGG reduces local and systemic myeloid-driven immunosuppression and enhances immune-mediated anti-tumor responses against GB [32].
CCL18	3.634	0.00095	- CCR binding - Chemokine	0.019*	CCL18 has been identified as a critical driver of GB malignant behaviors [33].
CLEC7A	1.4	0.00172	- (1->3)-beta-D-glucan binding - Carbohydrate binding - Identical protein binding - Metal ion binding - MHC binding - PRR activity	0.04*	CLEC7A upregulation was related to poorer GB prognosis and more APCs infiltration [34]. ***
LTBR	1.178	0.00213	- Identical protein binding - Kinase binding - Posphatase binding - Thioesterase binding - Tumor necrosis factor receptor binding - Ubiquitin protein ligase and transferase - Zinc ion binding	0.041*	Low expression of LTBR indicates good prognosis [35]. ***
IL1B	1.785	0.00216	- Cytokine - Integrine binding - IL1R binding - Protein domain specific binding	0.15	High expression of IL1B in GB patients have a significant impact for low OS [36]. ***
THBD	1.486	0.00289	- Calcium ion binding - Transmembrane signaling receptor	0.041*	THBD increased TMZ resistance and glycolysis in TMZ-resistant cells [37].
NCF4	1.158	0.00514	- PI3P binding - Superoxide-generating NADPH oxidase activator activity	0.12	Diminished NCF4 RNA expression is significantly associated with increased glioma survival [38]. ***
ALOX5	1.288	0.0057	- Arachidonate 12(S)//5//8(S)-lipoxygenase - Hydrolase - Iron ion binding	0.0068***	Inhibition of the lipoxygenase pathway has been shown to induce apoptosis in a variety of cancer cells [39].
LRG1	1.813	0.0057	- Type I/II transforming growth factor beta receptor binding	0.031*	Silencing the expression of LRG1 suppresses the growth of GB U251 cells in vitro and in vivo [40]. ***
CCL23	2.379	0.0057	- CCR binding - Chemokine - Heparin binding	0.045*	no relevant info
CCL4L2	2.406	0.00577	- CCR binding - Chemokine	0.11	no relevant info
CCL7	3.285	0.00668	- CCR1/2 binding - Chemokine - Heparin binding	0.031*	High expression of CCL7 is associated with negative prognostic outcomes in GB patients [41]. ***
CD33	1.146	0.0083	- Carbohydrate binding - Phosphatase binding - Sialic acid binding - Signaling receptor	0.35	CD33 (myeloid-derived suppressor cell) levels are highly positively correlated with nCBV values [42].
TLR2	1.227	0.0083	- Amyloid-beta binding - LPS immune receptor - NADP + nucleosidase - PRR activity - Peptidoglican binding - Protein-containing complex binding - Transmembrane signaling Toll-like receptor - Triacyl lipopeptide binding	0.049*	TLR2 has been identified as the main TLR controlling microglial MT1-MMP expression and promoting glioma expansion [43].
SAA1	2.919	0.00854	- G-protein coupled receptor - Heparin binding	0.0037***	High expression of SAA1 is associated with poor survival in GB [44]. ***
NR4A2	1.328	0.00978	- Beta-catenin binding - Transcription activator of RNA polymerase II - Nuclear glucocorticoid receptor - Nuclear retinoid X receptor - Zinc ion binding - Heterodimerization	0.045*	NR4A2 is pro-oncogenic in HGG [45].
LILRA5	1.892	0.00978	- Inhibitory MHC class I receptor	0.019*	no relevant info

**Fig. 4:**
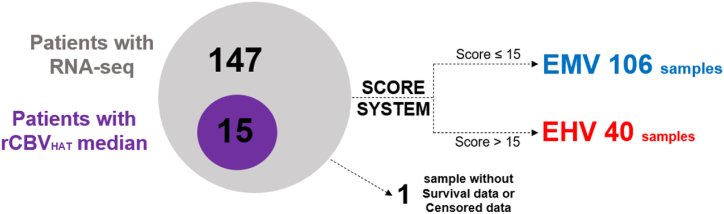
Diagram explaining how the EMV (Expected Moderate Vascularity Group) and EHV (Expected High Vascularity Group) were defined according to the Score system generated from the 31 genes differentially expressed in the DGE analysis between the HV and -V groups.

### 3.5 DGE correlation with prognosis – Kaplan-Meier curves and log-rank test

Two groups were defined based on the genes overexpressed in the HV group compared to the -V group in the DGE analysis, using the Score system (Fig. 4). The cut-off of 15 was defined to include the patients in either the EMV or EHV group. The EHV group included 40 patients, of whom 35 had valid data for the Log-Rank test, while the EMV group included 106 patients, of whom 84 had valid data. Only one patient was excluded due to a lack of survival data. The median survival times for the EMV and EHV groups were found to be 358 and 290 days, respectively.

**Fig. 5:**
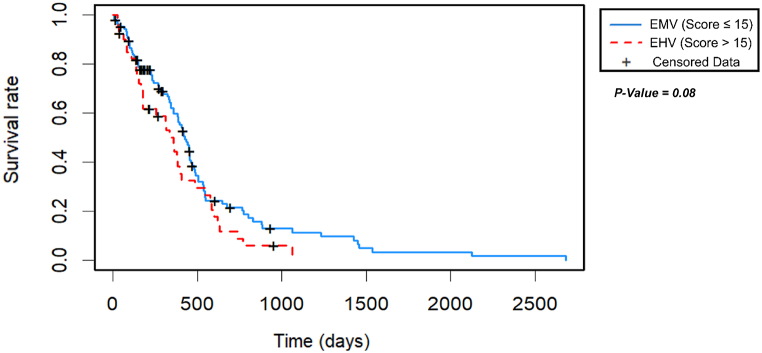
Results of the Kaplan-Meier curves for the most significative cut-off (cut-off = 15) in Score system using 21 overexpressed genes in HV group. *P*-value of the log-rank test associated with these curves = 0.084. The blue curve shows prognostic data for 106 patients, 22 of which are censored data (represented by crosses). The red curve shows prognostic data for 40 patients, 5 of them are censored data. (For interpretation of the references to colour in this figure legend, the reader is referred to the Web version of this article.)

Overexpression of genes associated with macroscopic vascularity is associated with a worse prognosis according to Kaplan-Meier curves in Fig. 5. We can see how in the first days, the differences between the EHV group (red line, score >15) and EMV group (blue line, score ≤15), are practically identical. However, after 200 days, the prognosis in the EHV group, and therefore the expected low prognosis, declines more rapidly than in the EMV group. The Log Rank results (p-value = 0.08) confirmed the significant difference between the two Kaplan-Meier curves.

## 4 Discussion

The current study has yielded a gene set that had a significant association with high vascularity defined by non-invasive MRI parameters in GB. Additionally, this gene set was linked to poor prognosis in the patient cohort.

In a study conducted on THPA [46], researchers conducted survival analyses using the TCGA-GBM sample. The key difference between the THPA's research and ours is in the approach to finding correlations with survival. The THPA's investigation was dedicated to discovering correlations between individual genes and survival, whereas our study revealed a correlation with survival through the set of genes acquired from our differential gene expression analysis. Our results showed that 13 out of 21 genes associated with worse prognosis were in agreement with THPA's findings. Some of them, including CCL4, IL10, and THBD, have well-studied mechanisms that contribute to tumor growth promotion. The release of CCL4 aids in tumor invasion [30], IL10 plays a role in immunosuppression [31], and THBD contributes to enhanced resistance to TMZ [37]. This reinforces the potential of this angiogenic and pro-tumoral vascularization gene set itself as a prognosis biomarker. In addition, a worse prognosis correlated with the overexpression of 16 of our genes has also been demonstrated in several studies in the literature [[30], [31], [32], [33], [34], [35], [36], [37], [38], [39], [40], [41], [42], [43], [44], [45]]. Consequently, our gene set findings provide validation for the notion that higher tumor vascularity corresponds to a more unfavorable prognosis, reinforcing the importance of investigating tumor vascularity. Our gene set results not only validate these findings but also underscore the significance of investigating lesser-studied genes in the literature, highlighting their potential value in advancing research in this field.

We did not find differential expression in the MV vs -V analysis. To address this, hypotheses could be proposed regarding the assignment of a lower rCBV_HAT_ median threshold, so as to obtain a group of samples with lower vascularity than ours, and to be able to glimpse some difference at the transcriptomic level with the group of unknown vascularity.

The Kaplan-Meier curves (based on a supervised analysis) are aligned with validated survival biomarkers for glioblastoma. Within the first 200 days, it seems that there are more significant determining factors than high tumor progression: age at diagnosis [47], tumor location [48], response to treatment, or extent of surgical resection, among others. However, after 200 days, the survival curves of our Score system diverge significantly. The prognostic marker rCBV_HAT_ median has already been shown to diverge and be significant past these 200 days in other studies [21,22]. Thus, it is crucial to emphasize the strong association between tumor progression and vascularization beyond this stage of diagnosis.

Two significant limitations were present in the study. Firstly, the sample size of patients with MRI-defined vascularity was limited. Secondly, the use of an equal weighting system for all genes in the score system potentially overlooked vital contributions from individual genes [49]. Addressing these limitations through further validation studies could refine the accuracy and applicability of the findings, informing future clinical approaches.

## 5 Conclusion & recommendations

The results of this study demonstrate a strong association between the expression of specific genes and increased macroscopic vascularity in glioblastoma patients, as determined by the rCBV at HAT habitat from perfusion-weighted MRI images. Additionally, our findings indicate that this gene profile is linked to poor prognosis in the patient cohort. This in silico study not only establishes working hypotheses connecting specific gene sets to the macroscopic vascularity observed on MRI but also highlights the discovery of genes related to MRI vascularity that have not been previously studied in the literature. These findings reveal complex and previously unrecognized multilevel relationships in glioblastoma biology, underscoring the novel aspects of our research [50]. The validation of these results by previous studies suggests the potential value of these genes as prognosis biomarkers for GB. Further validation studies with larger sample sizes and more precise gene weighting systems should be conducted to confirm the potential of these genes as biomarkers for GB. Moreover, there is a pressing need for further investigation to underscore the prognostic significance of the identified gene set in more extensive cohorts. Finally, the integration of gene expression profiles into clinical practice should involve the development of tests to rapidly and accurately measure their expression levels in tumor samples, thus establishing a complete panel of prognostic genetic biomarkers associated with certain MR perfusion gene expression values, leading to a more accurate, inexpensive, and non-invasive assessment of patients with GB [51].

## Availability of data and materials

The MRI data that support the findings of this study are openly available at The Cancer Imaging Atlas at https://doi.org/10.7937/K9/TCIA.2016.RNYFUYE9. Access to the images processed by ONCOhabitats is available on demand on Zenodo at https://doi.org/10.5281/zenodo.4704090. Access to RNA-seq data is openly available at The Cancer Genome Atlas at: https://portal.gdc.cancer.gov/projects/TCGA-GBM.

## Ethics approval and consent to participate

All the data used in this article is publicly available, being waived ethical and consent approval.

## Consent for publication

All the authors consent the publication of this study.

## CRediT authorship contribution statement

**Víctor Montosa-i-Micó:** Writing – review & editing, Writing – original draft, Methodology, Investigation, Formal analysis, Data curation, Conceptualization. **María del Mar Álvarez-Torres:** Writing – review & editing, Writing – original draft, Supervision, Investigation, Data curation, Conceptualization. **Rebeca Burgos-Panadero:** Writing – review & editing, Validation, Supervision. **F. Javier Gil-Terrón:** Formal analysis, Data curation. **Maria Gómez Mahiques:** Writing – review & editing, Methodology. **Carles Lopez-Mateu:** Writing – review & editing, Methodology. **Juan M. García-Gómez:** Writing – review & editing, Writing – original draft, Supervision, Conceptualization. **Elies Fuster-Garcia:** Writing – review & editing, Writing – original draft, Supervision, Methodology, Formal analysis, Conceptualization.

## Declaration of competing interest

The authors declare that they have no known competing financial interests or personal relationships that could have appeared to influence the work reported in this paper.

## Abbreviations

GB: Glioblastoma
MRI: Magnetic Resonance Imaging
DSC: Dynamic Susceptibility Contrast
RNA-seq: RNA Sequencing
TCGA-GBM: The Cancer Genome Atlas - Glioblastoma Multiforme
CNS: Central Nervous System
rCBV: Relative Cerebral Blood Volume
TCIA: The Cancer Imaging Archive
GBCA: Gadolinium-Based Contrast Agent
CNN: Convolutional Neural Network
CBF: Cerebral Blood Flow
MTT: Mean Transit Time
CBV: Cerebral Blood Volume
HAT: High-Angiogenic Tumor
LAT: Low-Angiogenic Tumor
IPE: Infiltrated Peripheral Edema
VPE: Vasogenic Peripheral Edema
HV: High Vascularity group
MV: Moderate Vascularity group
-V: Unknown Vascularity group
GO: Gene Ontology
CPM: Counts per Million
OS: Overall Survival
EHV: Expected High Vascularity
EMV: Expected Moderate Vascularity
THPA: The Human Protein Atlas
CCL4: C–C Motif Chemokine Ligand 4
CCL3: C–C Motif Chemokine Ligand 3
IL10: Interleukin-10
C5AR2: Complement C5a Receptor 2
IL6: Interleukin-6
CCL18: C–C Motif Chemokine Ligand 18
CLEC7A: C-Type Lectin Domain Containing 7A
LTBR: Lymphotoxin Beta Receptor
IL1B: Interleukin-1 Beta
THBD: Thrombomodulin
NCF4: Neutrophil Cytosolic Factor 4
ALOX5: Arachidonate 5-Lipoxygenase
LRG1: Leucine-Rich Alpha-2-Glycoprotein 1
CCL23: C–C Motif Chemokine Ligand 23
CCL4L2: C–C Motif Chemokine Ligand 4-Like 2
CCL7: C–C Motif Chemokine Ligand 7
CD33: Cluster of Differentiation 33
TLR2: Toll-Like Receptor 2
SAA1: Serum Amyloid A1
NR4A2: Nuclear Receptor Subfamily 4 Group A Member 2
LILRA5: Leukocyte Immunoglobulin-Like Receptor A5
CCR: C–C Chemokine Receptor
MHC: Major Histocompatibility Comple
PRR: Pattern Recognition Receptor
LPS: Lipopolysaccharide
