# Supplementary material for: The prognostic relevance of a gene expression signature in MRI-defined highly vascularized glioblastoma
Source: Heliyon. 2024 May 17;10(11):e31175. doi: 10.1016/j.heliyon.2024.e31175 (PMC11145239; doi:10.1016/j.heliyon.2024.e31175)
Supplement: Multimedia component 1 [file mmc1.docx]

**Additional file 1: TCGA Case IDs and Sample IDs**

| LOW VASCULARITY GROUP |  |  | Case ID | Sample ID |
| --- | --- | --- | --- | --- |
|  |  |  | TCGA-76-4926 | TCGA-76-4926-01B |
| HIGH VASCULARITY GROUP |  |  | TCGA-27-1834 | TCGA-27-1834-01A |
|  |  |  | TCGA-41-5651 | TCGA-41-5651-01A |
| UNDEFINED VASCULARITY GROUP | |  | TCGA-27-1832 | TCGA-27-1832-01A |
|  |  |  | TCGA-12-3653 | TCGA-12-3653-01A |
|  |  |  | TCGA-06-2561 | TCGA-06-2561-01A |
| Case ID | Sample ID |  | TCGA-28-5213 | TCGA-28-5213-01A |
| TCGA-06-0184 | TCGA-06-0184-01A |  | TCGA-26-5139 | TCGA-26-5139-01A |
| TCGA-06-0644 | TCGA-06-0644-01A |  | TCGA-27-1835 | TCGA-27-1835-01A |
| TCGA-06-0178 | TCGA-06-0178-01A |  | TCGA-06-0219 | TCGA-06-0219-01A |
| TCGA-06-0130 | TCGA-06-0130-01A |  | TCGA-06-2562 | TCGA-06-2562-01A |
| TCGA-06-0878 | TCGA-06-0878-01A |  | TCGA-06-5414 | TCGA-06-5414-01A |
| TCGA-06-0174 | TCGA-06-0174-01A |  | TCGA-32-1982 | TCGA-32-1982-01A |
| TCGA-06-0168 | TCGA-06-0168-01A |  | TCGA-06-0743 | TCGA-06-0743-01A |
| TCGA-06-0187 | TCGA-06-0187-01A |  | TCGA-06-0210 | TCGA-06-0210-01A |
| TCGA-06-0238 | TCGA-06-0238-01A |  | TCGA-19-2619 | TCGA-19-2619-01A |
| TCGA-06-0645 | TCGA-06-0645-01A |  | TCGA-19-2625 | TCGA-19-2625-01A |
| TCGA-06-0141 | TCGA-06-0141-01A |  | TCGA-02-0047 | TCGA-02-0047-01A |
| TCGA-06-0646 | TCGA-06-0646-01A |  | TCGA-14-0781 | TCGA-14-0781-01B |
| TCGA-06-0138 | TCGA-06-0138-01A |  | TCGA-06-2567 | TCGA-06-2567-01A |
| TCGA-06-0139 | TCGA-06-0139-01A |  | TCGA-12-5295 | TCGA-12-5295-01A |
| TCGA-06-0190 | TCGA-06-0190-01A |  | TCGA-32-5222 | TCGA-32-5222-01A |
| TCGA-06-5859 | TCGA-06-5859-01A |  | TCGA-19-1787 | TCGA-19-1787-01B |
| TCGA-27-2524 | TCGA-27-2524-01A |  | TCGA-28-5208 | TCGA-28-5208-01A |
| TCGA-15-0742 | TCGA-15-0742-01A |  | TCGA-28-5204 | TCGA-28-5204-01A |
| TCGA-26-5136 | TCGA-26-5136-01B |  | TCGA-32-4213 | TCGA-32-4213-01A |
| TCGA-12-3650 | TCGA-12-3650-01A |  | TCGA-08-0386 | TCGA-08-0386-01A |
| TCGA-27-2526 | TCGA-27-2526-01A |  | TCGA-28-5215 | TCGA-28-5215-01A |
| TCGA-32-2638 | TCGA-32-2638-01A |  | TCGA-06-2565 | TCGA-06-2565-01A |
| TCGA-06-0156 | TCGA-06-0156-01A |  | TCGA-28-2514 | TCGA-28-2514-01A |
| TCGA-06-5412 | TCGA-06-5412-01A |  | TCGA-28-2509 | TCGA-28-2509-01A |
| TCGA-02-2485 | TCGA-02-2485-01A |  | TCGA-19-4065 | TCGA-19-4065-01A |
| TCGA-14-1825 | TCGA-14-1825-01A |  | TCGA-28-5218 | TCGA-28-5218-01A |
| TCGA-32-2615 | TCGA-32-2615-01A |  | TCGA-06-0745 | TCGA-06-0745-01A |
| TCGA-27-2519 | TCGA-27-2519-01A |  | TCGA-32-2634 | TCGA-32-2634-01A |
| TCGA-41-2572 | TCGA-41-2572-01A |  | TCGA-06-2569 | TCGA-06-2569-01A |
| TCGA-02-0055 | TCGA-02-0055-01A |  | TCGA-32-1980 | TCGA-32-1980-01A |
| TCGA-28-2499 | TCGA-28-2499-01A |  | TCGA-27-1830 | TCGA-27-1830-01A |
| TCGA-76-4932 | TCGA-76-4932-01A |  | TCGA-26-5132 | TCGA-26-5132-01A |
| TCGA-06-0125 | TCGA-06-0125-01A |  | TCGA-76-4927 | TCGA-76-4927-01A |
| TCGA-28-5209 | TCGA-28-5209-01A |  | TCGA-12-1597 | TCGA-12-1597-01B |
| TCGA-76-4925 | TCGA-76-4925-01A |  | TCGA-26-5133 | TCGA-26-5133-01A |
| TCGA-06-0157 | TCGA-06-0157-01A |  | TCGA-06-0211 | TCGA-06-0211-01A |
| TCGA-28-2510 | TCGA-28-2510-01A |  | TCGA-06-0747 | TCGA-06-0747-01A |
| TCGA-14-0817 | TCGA-14-0817-01A |  | TCGA-76-4928 | TCGA-76-4928-01B |
| TCGA-14-0871 | TCGA-14-0871-01A |  | TCGA-19-1390 | TCGA-19-1390-01A |
| TCGA-41-3915 | TCGA-41-3915-01A |  | TCGA-41-2571 | TCGA-41-2571-01A |
| TCGA-06-2559 | TCGA-06-2559-01A |  | TCGA-06-5416 | TCGA-06-5416-01A |
| TCGA-16-0846 | TCGA-16-0846-01A |  | TCGA-14-2554 | TCGA-14-2554-01A |
| TCGA-06-5408 | TCGA-06-5408-01A |  | TCGA-06-5858 | TCGA-06-5858-01A |
| TCGA-19-2620 | TCGA-19-2620-01A |  | TCGA-19-5960 | TCGA-19-5960-01A |
| TCGA-12-3652 | TCGA-12-3652-01A |  | TCGA-26-5135 | TCGA-26-5135-01A |
| TCGA-02-2486 | TCGA-02-2486-01A |  | TCGA-06-0750 | TCGA-06-0750-01A |
| TCGA-27-1837 | TCGA-27-1837-01A |  | TCGA-12-0618 | TCGA-12-0618-01A |
| TCGA-12-0619 | TCGA-12-0619-01A |  | TCGA-14-0790 | TCGA-14-0790-01B |
| TCGA-12-0821 | TCGA-12-0821-01A |  | TCGA-27-1831 | TCGA-27-1831-01A |
| TCGA-14-1034 | TCGA-14-1034-01A |  | TCGA-06-0744 | TCGA-06-0744-01A |
| TCGA-28-2513 | TCGA-28-2513-01A |  | TCGA-16-1045 | TCGA-16-1045-01B |
| TCGA-32-1970 | TCGA-32-1970-01A |  | TCGA-27-2528 | TCGA-27-2528-01A |
| TCGA-28-5207 | TCGA-28-5207-01A |  | TCGA-28-5216 | TCGA-28-5216-01A |
| TCGA-19-2624 | TCGA-19-2624-01A |  | TCGA-06-5410 | TCGA-06-5410-01A |
| TCGA-06-0158 | TCGA-06-0158-01A |  | TCGA-06-5411 | TCGA-06-5411-01A |
| TCGA-06-2564 | TCGA-06-2564-01A |  | TCGA-06-1804 | TCGA-06-1804-01A |
| TCGA-06-5413 | TCGA-06-5413-01A |  | TCGA-28-1753 | TCGA-28-1753-01A |
| TCGA-14-1829 | TCGA-14-1829-01A |  | TCGA-15-1444 | TCGA-15-1444-01A |
| TCGA-27-2523 | TCGA-27-2523-01A |  | TCGA-28-1747 | TCGA-28-1747-01C |
| TCGA-76-4929 | TCGA-76-4929-01A |  | TCGA-76-4931 | TCGA-76-4931-01A |
| TCGA-06-0132 | TCGA-06-0132-01A |  | TCGA-32-2616 | TCGA-32-2616-01A |
| TCGA-14-0787 | TCGA-14-0787-01A |  | TCGA-12-5299 | TCGA-12-5299-01A |
| TCGA-28-5220 | TCGA-28-5220-01A |  | TCGA-06-2557 | TCGA-06-2557-01A |
| TCGA-26-5134 | TCGA-26-5134-01A |  | TCGA-06-2558 | TCGA-06-2558-01A |
| TCGA-32-2632 | TCGA-32-2632-01A |  | TCGA-06-0749 | TCGA-06-0749-01A |
| TCGA-12-0616 | TCGA-12-0616-01A |  | TCGA-14-0789 | TCGA-14-0789-01A |
| TCGA-41-4097 | TCGA-41-4097-01A |  | TCGA-06-0649 | TCGA-06-0649-01B |
| TCGA-06-0686 | TCGA-06-0686-01A |  | TCGA-06-0882 | TCGA-06-0882-01A |
| TCGA-06-5418 | TCGA-06-5418-01A |  | TCGA-06-2563 | TCGA-06-2563-01A |
| TCGA-14-1823 | TCGA-14-1823-01A |  | TCGA-06-5856 | TCGA-06-5856-01A |
