# Supplementary material for: The prognostic relevance of a gene expression signature in MRI-defined highly vascularized glioblastoma
Source: Heliyon. 2024 May 17;10(11):e31175. doi: 10.1016/j.heliyon.2024.e31175 (PMC11145239; doi:10.1016/j.heliyon.2024.e31175)
Supplement: Multimedia component 2 [file mmc2.docx]

**Additional file 2:**

***Differential gene expression model - Voom plot***

Figure S1 shows the voom plot results needed to build the regression models used in gene expression analysis. With voom plot it can be verified wether our pipeline draws the characteristic curve of an RNA-seq analysis model, since the counts have a characteristic distribution in which the mean and the variance are related. The relationship between counts and variability is as expected in an analysis of RNA-seq samples: counts are inversely proportional to variability. The red line that shows the mean variance trend presents the characteristic curves of an RNA-seq analysis model.


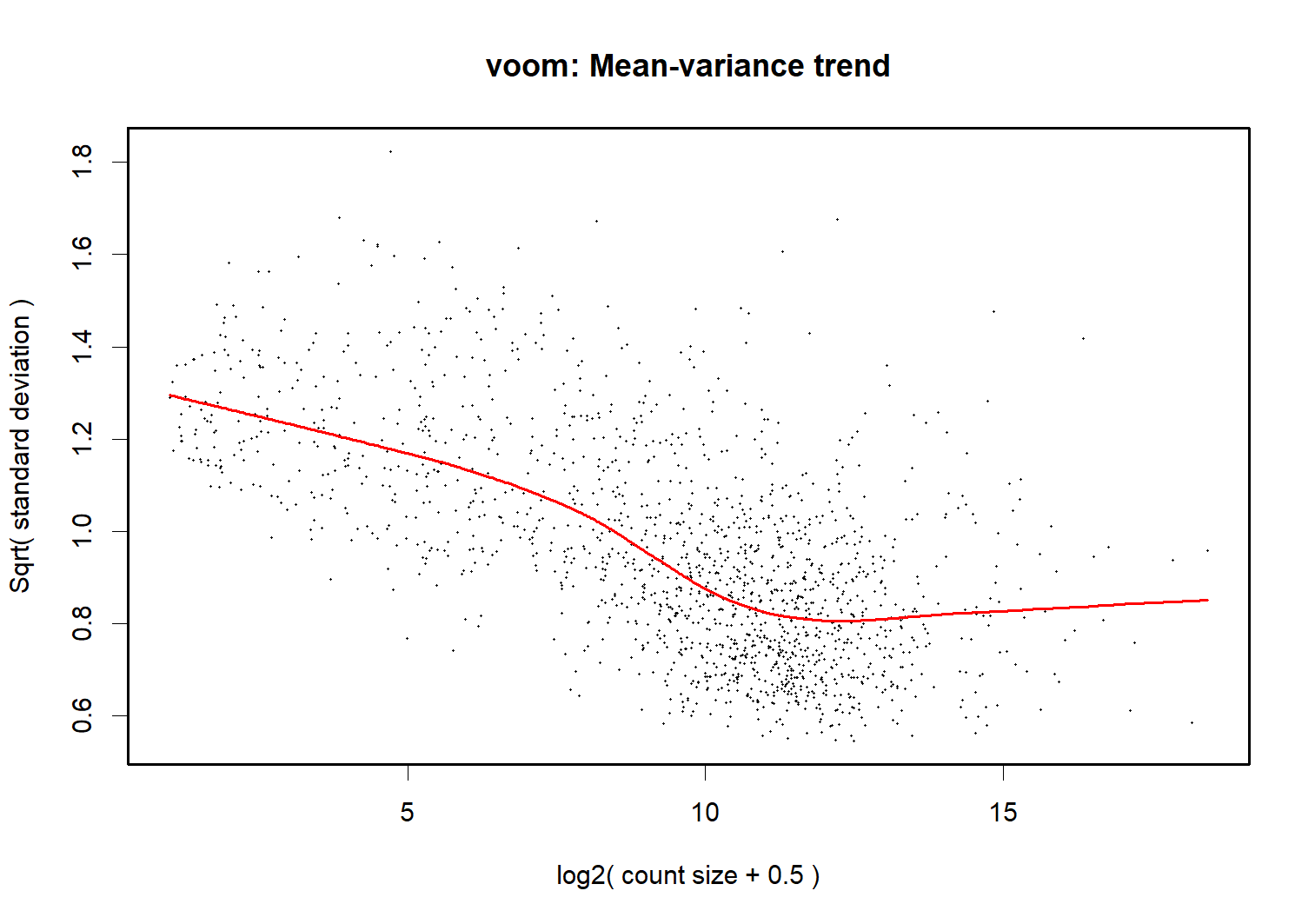


Figure S1. Voom plot. The graph shows the mean variance of the counts. The red line draws the mean variance trend.
